# A New Anticancer Semisynthetic Theobromine Derivative Targeting EGFR Protein: CADDD Study

**DOI:** 10.3390/life13010191

**Published:** 2023-01-09

**Authors:** Ibrahim H. Eissa, Reda G. Yousef, Hazem Elkady, Aisha A. Alsfouk, Bshra A. Alsfouk, Dalal Z. Husein, Ibrahim M. Ibrahim, Eslam B. Elkaeed, Ahmed M. Metwaly

**Affiliations:** 1 Pharmaceutical Medicinal Chemistry & Drug Design Department, Faculty of Pharmacy (Boys), Al-Azhar University, Cairo 11884, Egypt; 2 Department of Pharmaceutical Sciences, College of Pharmacy, Princess Nourah bint Abdulrahman University, Riyadh 11671, Saudi Arabia; 3 Chemistry Department, Faculty of Science, New Valley University, El-Kharja 72511, Egypt; 4 Biophysics Department, Faculty of Science, Cairo University, Cairo 12613, Egypt; 5 Department of Pharmaceutical Sciences, College of Pharmacy, AlMaarefa University, Riyadh 13713, Saudi Arabia; 6 Pharmacognosy and Medicinal Plants Department, Faculty of Pharmacy (Boys), Al-Azhar University, Cairo 11884, Egypt; 7 Biopharmaceutical Products Research Department, Genetic Engineering and Biotechnology Research Institute, City of Scientific Research and Technological Applications (SRTA-City), Alexandria 21934, Egypt

**Keywords:** EGFR inhibitor, semi synthesis, CADDD, DFT, docking, MD simulations, anticancer

## Abstract

A new lead compound has been designed as an antiangiogenic EGFR inhibitor that has the pharmacophoric characteristics to bind with the catalytic pocket of EGFR protein. The designed lead compound is a (para-chloro)acetamide derivative of the alkaloid, theobromine, (**T-1-PCPA**). At first, we started with deep density functional theory (DFT) calculations for **T-1-PCPA** to confirm and optimize its 3D structure. Additionally, the DFT studies identified the electrostatic potential, global reactive indices and total density of states expecting a high level of reactivity for **T-1-PCPA**. Secondly, the affinity of **T-1-PCPA** to bind and inhibit the EGFR protein was studied and confirmed through detailed structure-based computational studies including the molecular docking against EGFR^WT^ and EGFR^T790M^, Molecular dynamics (MD) over 100 ns, MM-GPSA and PLIP experiments. Before the preparation, the computational ADME and toxicity profiles of **T-1-PCPA** have been investigated and its safety and the general drug-likeness predicted. Accordingly, **T-1-PCPA** was semi-synthesized to scrutinize the proposed design and the obtained in silico results. Interestingly, **T-1-PCPA** inhibited in vitro EGFR^WT^ with an IC_50_ value of 25.35 nM, comparing that of erlotinib (5.90 nM). Additionally, **T-1-PCPA** inhibited the growth of A549 and HCT-116 malignant cell lines with IC_50_ values of 31.74 and 20.40 µM, respectively, comparing erlotinib that expressed IC_50_ values of 6.73 and 16.35 µM, respectively.

## 1. Introduction

According to estimates from the World Health Organization (WHO), cancer ranks globally second as a cause of death after cardiovascular diseases [1]. Cancer therapy is a challenging area for medicinal chemistry scientists to discover safe and effective targeted chemotherapeutic agents that fight cancer’s growth through the inhibition of specific molecular targets [2]. It is well-known that tumor development as well as reproduction are highly linked to the increased vascularity (angiogenesis) in the malignant cells, so utilizing anti-angiogenesis mechanisms is a potential strategy to combat cancer [3]. EGFR plays a key role in angiogenesis and cancer cell growth [4]. Cellular proliferation, differentiation and survival are enhanced when EGFR is overexpressed [5]. Multiple types of cancer have elevated EGFR levels, which promote solid tumor growth. A high level of expression of EGFR has been linked to a reduced survival rate across several cancer types, and this expression acts as a strong prognostic indicator [6]. The overexpression of EGFR receptors in cancer cells also enabled researchers to target them as an anticancer strategy [7].

The computer-aided drug design and discovery (CADDD) is a fast growing scientific area that was very successful in the past few years [8,9]. Many pharmaceutical companies [10], as well as academics [11,12], have depended on the CADDD in the area of drug discovery. The accelerated advancements in structural bio-informatics, genomics and proteomics auspiciously led the efforts toward a modern-era of drug discovery. Enormous researches over the last decades utilized the docking algorithms to predict the activities of certain molecules [13,14]. Also, several docking programs have been created and employed to analyze the binding process between a certain molecule and an examined receptor [15]. Molecular Docking could analyze that interaction through the identification of binding energies as well as binding modes of the docked molecules inside the active sites of the targeted receptors [16]. Over the last few years, our team has applied the CADDD in the design and synthesis of several anti-EGFR compounds belonging to pyrimidine [17,18,19] and xanthin [20] derivatives.

### Rationale

**Figure 1:**
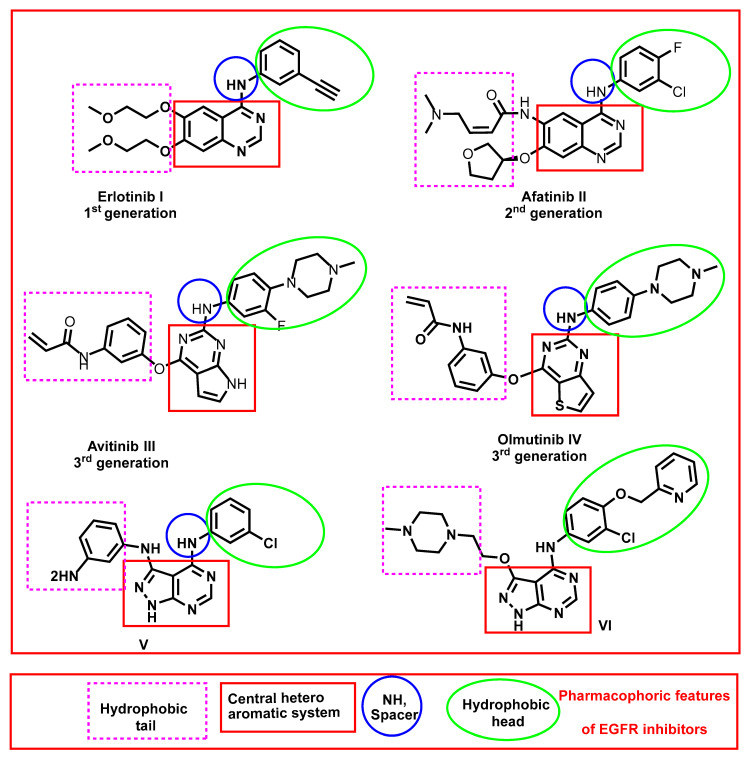
The chemical structures (showing the essential features) of some reported anti-EGFR drugs.

The 1st generation of the FDA-approved EGFR inhibitors (as erlotinib **I** [21]) showed very strong activities against the non-small-cell type of lung cancer. Unfortunately, the 1st generation drugs exhibited a mutation-related resistance that has been resolved later by the 2nd generation of EGFR inhibitors (such as afatinib **II** [22]). The 3rd generation of anti-EGFR drugs (such as avitinib **III** [23] and olmutinib **IV** [24]) was approved to overcome the 2nd generation’s induced toxicity, showing enhanced activities against EGFR^T790M^ than EGFR^WT^. Additionally, some derivatives of 1*H*-pyrazolo [3,4-*d*]pyrimidine as compounds **V**, *N*^3^-(3-aminophenyl)-*N*^4^-(3-chlorophenyl)-1*H*-pyrazolo [3,4-*d*]pyrimidine-3,4-diamine, [25] and **VI**, *N*-Me-piperazinyl derivative of 4-anilino-1*H*-pyrazolo [3,4-*d*]pyrimidine, [26] exhibited good inhibitory effects against EGFR. As illustrated in Figure 1**,** four pharmacophoric features were found in EGFR inhibitor drugs. These four features are crucial to the fitting against the EGFR’s active site [27]. These features are a central hetero aromatic system, an NH spacer, a hydrophobic head, as well as a hydrophobic tail. These functional groups occupy and bind with the adenine binding pocket, the linker region, hydrophobic regions I and II, respectively, inside the EGFR’s active site [28,29,30].

**Figure 2:**
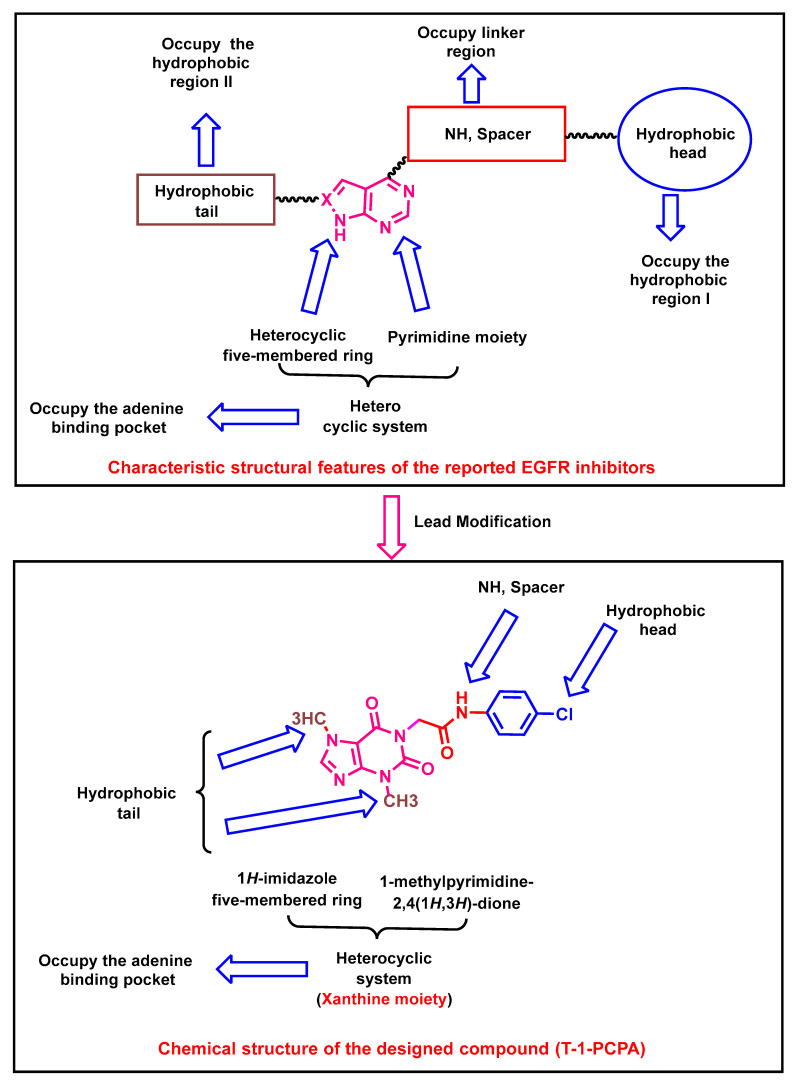
The rationale of **T-1-PCPA** design.

In ancient historical as well as in recent records, nature provided human beings with their primary needs, such as treatment, food and cosmetical products [31,32]. Accordingly, as shown in Figure 2, we employed the natural alkaloid, theobromine, to obtain the lead compound, **T-1-PCPA,** (*N*-(4-Chlorophenyl)-2-(3,7-dimethyl-2,6-dioxo-2,3,6,7-tetrahydro-1*H*-purin-1-yl)acetamide)**. T-1-PCPA** has acetamide, p-clorobenzene and methyl moieties representing the hetero central aromatic system, NH spacer, hydrophobic head and tail, respectively. In detail, the xanthine moiety works as a heterocyclic system to occupy the adenine binding pocket. The xanthine moiety comprises a pyrimidine and 1H-imidazole rings. Accordingly, the xanthine moiety has high similarity with the adenine moiety of the ATP. The high similarity may encourage the competitive inhibition at the ATP binding site. Furthermore, the essential NH spacer was included in acetamide moiety to increase the flexibility and affinity against the ATP binding site. In addition, to occupy hydrophobic region I, the p-chlorophenyl moiety was utilized. The high hydrophobicity of both phenyl ring and the chloro atom may enhance the stability of the designed compound at the active pocket via many hydrophobic interactions. Finally, the two methyl moieties at positions 3- and 7-s of xanthin represented the hydrophobic tail to interact with the hydrophobic region II. The hydrophobic interactions of these two methyl groups can stabilize the designed molecule at the active site.

## 2. Results

### 2.1. DFT Studies

#### 2.1.1. Geometry Optimization and Mulliken Charge

**Figure 3:**
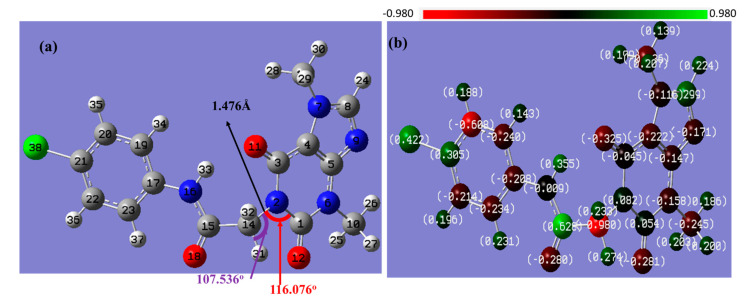
The fully optimized chemical structure (**a**), and the (**b**) Mulliken charge of **T-1-PCPA** at B3LYB/6-311G++(d, p) level.

The designed molecule, **T-1-PCPA**, was investigated and optimized at level B3LYP/6-311++G(d, p), Figure 3a. According to the semi-synthesis plan, **T-1-PCPA** should be obtained from the reaction of potassium 3,7-dimethyl-3,7-dihydro-1*H*-purine-2,6-dione and 2-chloro-*N*-(4-chlorophenyl) acetamide. The reaction between the two compounds should occur through the **C14-N2** bond (1.476 Å). The new two angles along the **C14-N2** bond were determined (Figure 3a). **T-1-PCPA** is a neutral singlet and consists of 164 electrons and 38 atoms. The optimized chemical structure of **T-1-PCPA** has a dipole moment of 8.625 Debye and a total ground state energy (TE) of −41,906.8 eV, as shown in Table 1. The value of dipole moment impacts the polarizability inside **T-1-PCPA** and, consequently, the transfer of charge wherein a larger value of dipole moment indicates a high biological reactivity. Another method for evaluating polarizability, molecular electrostatic potential and charge transfer is the Mulliken charge analysis. The color code of the charge distribution on the atoms of **T-1-PCPA** is shown in Figure 3b, wherein the most positively charged atoms are green and the most negatively charged atoms are red. According to the atomic charges, **C14** has the highest negative charge, and **C15** has the largest positive charge. Such a finding confirms the strong affinity and ease of charge transfer as an anticancer drug.

#### 2.1.2. Frontier Molecular Orbital (FMO) Analysis

**Figure 4:**
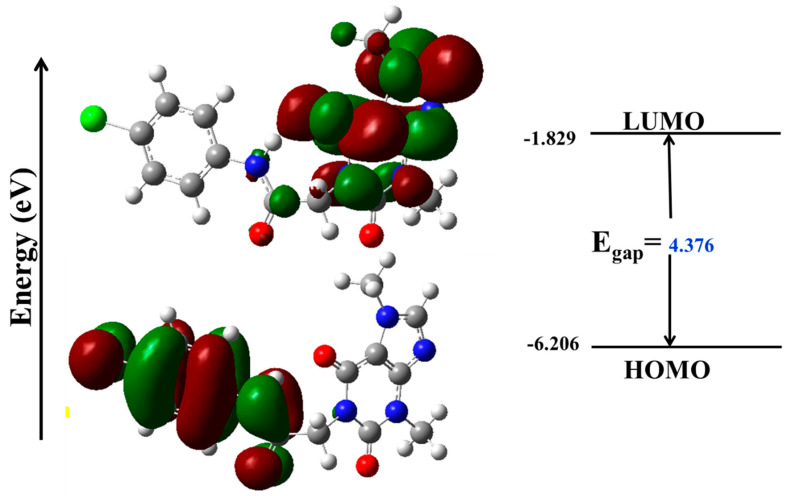
Molecular orbital surfaces and energy levels of **T-1-PCPA** by using DFT calculations at B3LYB/6-311G++(d, p) level.

The potential interaction sites within **T-1-PCPA** are disclosed by the FMO analysis. Figure 4 displays the isodensity maps of the HOMO and LUMO (FMO) orbitals. While the LUMO map shows that the purine moiety carries the largest electron density, the HOMO map shows that the electron density is focused on the acetamide moiety (the opposite terminal). Such an observation suggests that intra-molecular charge transfer inside **T-1-PCPA** is feasible. The sites that will be attacked by reactive agents are explained by the unique distribution of HOMO and LUMO over the molecule atoms, which ultimately represent the reactive character of **T-1-PCPA**. In order to investigate the electronic properties of **T-1-PCPA**, it is important to consider the energies of the FMOs (E_HOMO_ and E_LUMO_), as well as the energy difference between HOMO and LUMO (E_gap_). The computed E_gap_ value confirms the validity of electronic transitions inferring the presence of a reactive molecule [33]. The relevant metrics, including ionization potential (IP) and electron affinity (EA), are reported in Table 1. It is conducted that **T-1-PCPA** can obtain electrons because of its high electron affinity value.

#### 2.1.3. Chemical Reactivity Descriptors and Total Density of State (TDOS)

Numerous chemical reactivity descriptors, such as chemical hardness (η), chemical electronegativity (χ), global softness (δ), maximal charge acceptance (N_max_) and electrophilicity (ω), have been estimated using the FMO energy levels and E_gap_. Koopmans’ theory used to calculate these descriptors as described in the Supplementary Materials:

Electronegativity (χ) measures the drug’s ability to receive electrons, while hardness (η) measures the reverse, i.e., the stability. The extent of softness (σ) establishes the type of pharmacological reactivity. All calculated values in Table 1 confirm the expected drug’s inhibitory properties [34].

**Figure 5:**
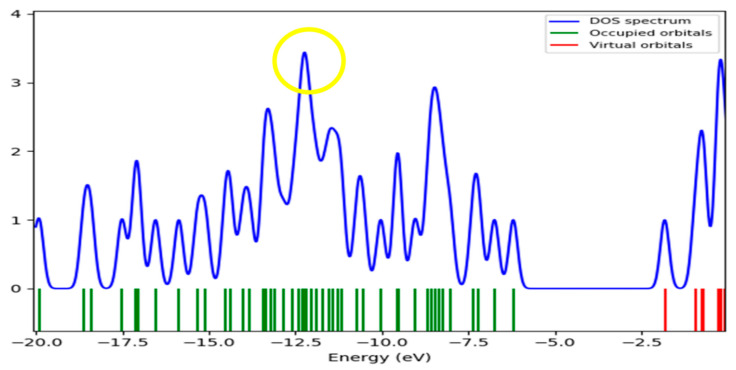
FMO spectrum and TDOS of **T-1-PCPA** at B3LYP/6-311++G(d, p).

Figure 5 shows the analysis and representation of “the total density distribution function, TDOS,” which is used to determine whether or not the FMO analysis provided a comprehensive electronic characterization. The TDOS spectrum of the prepared compound revealed that the greatest electronic density is located under the HOMO orbital.

#### 2.1.4. Electrostatic Potential (ESP) Maps

**Figure 6:**
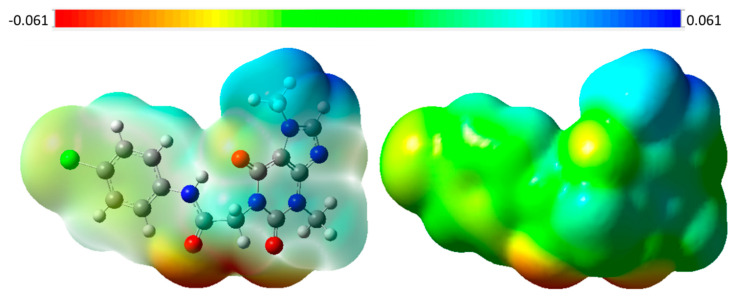
ESP maps of **T-1-PCPA** at the 6-311G++(d, p) level.

Electrostatic potential (ESP) is used mostly to propose active sites and their reactivities toward electrophilic and nucleophilic attacks, and it is related to molecular electron density. Figure 6 depicts the ESP maps, where blue regions (positive potentials) represent nucleophilic attack active sites, while red patches (negative potentials) represent electrophilic attack sites. Green and orange zones in the ESP color coding denote neutral and moderate potentials. In Figure 6, the green domain swelling is an indication of **T-1-PCPA**’s nucleophilic strength. The oxygen atoms have localized negative potentials that indicate proton-loving or electrophilic sites. Figure 6 shows that **T-1-PCPA** has potential and charge differences that support its viability as an anticancer medication.

### 2.2. Molecular Docking against EGFR

**Figure 7:**
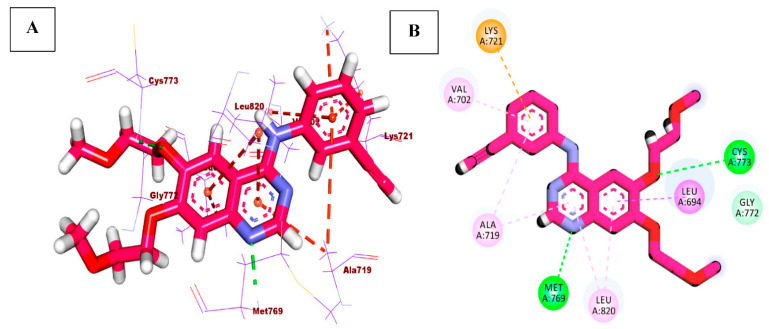
(**A**) 3D & (**B**) 2D interactions of erlotinib in EGFR^WT^.

The encouraging results of the DFT studies of **T-1-PCPA** inspired us to continue the research about that promising lead compound. The potential of **T-1-PCPA** to bind and inhibit the wild type of EGFR kinase (EGFR^WT^, PDB: 4HJO) was initially tested using the MOE software using Erlotinib, a standard EGFR inhibitor, as a reference. The docking pose of erlotinib revealed occupancy of the adenine pocket through essential hydrogen bond (HB) with Met769 and occupancy of the hydrophobic pocket in a hydrophobic network formed by Ala719, Val702 and Lys 721, which are shown in Figure 7**.**

**Figure 8:**
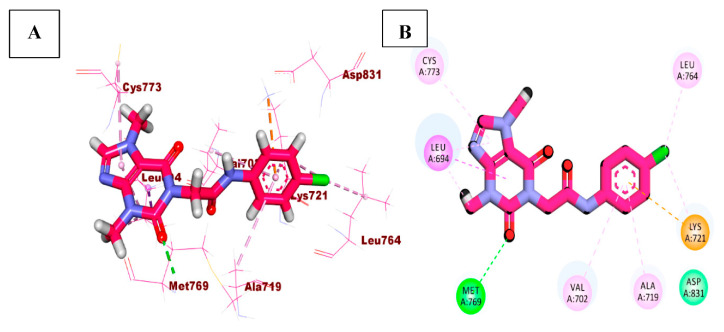
(**A**) 3D & (**B**) 2D interactions of **T-1-PCPA** in EGFR^WT^.

Interestingly, **T-1-PCPA** has a comparable binding mode to erlotinib. The purine arm was buried in the adenine pocket to form a key HB with Met769. Also, the 4-chlorophenyl arm was fixed in the hydrophobic pocket to form hydrophobic bonds with Ala719, Val702, Leu764 and Lys 721, as shown in Figure 8.

**Figure 9:**
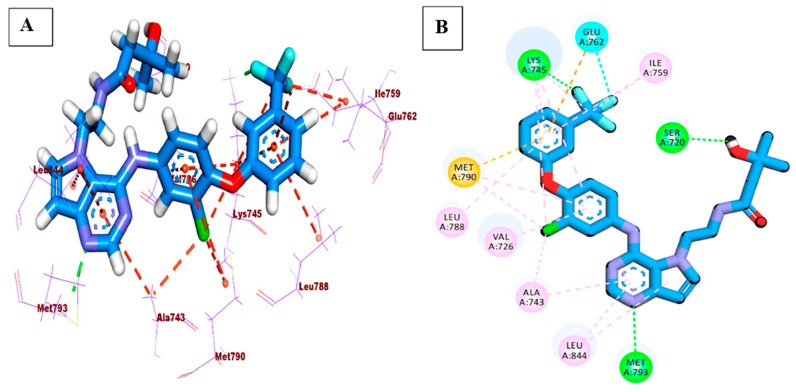
(**A**) 3D & (**B**) 2D interactions of TAK285 in EGFR^T790M^.

To support the previous findings, **T-1-PCPA** was docked against the mutant EGFR (EGFR^T790M^, PDB:3W2O) utilizing the co-crystallized ligand, TAK285, as a reference. TAK285 bonded to the EGFR^T790M^ active site revealing a key HB with Met793 in the adenine pocket and abundant hydrophobic bonds with Val726, Leu788, Ile759 and Lys745 in the hydrophobic pocket as illustrated in Figure 9.

**Figure 10:**
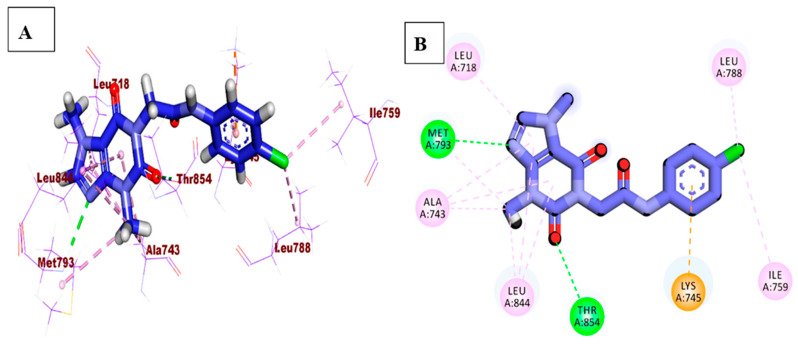
(**A**) 3D & (**B**) 2D interactions of **T-1-PCPA** in EGFR^T790M^.

It is attention-grabbing that T-1-PCPA has a binding mode nearly similar to TAK285. As presented in Figure 10**,** the purine and the 4-chlorophenyl moieties were buried in the adenine pocket (via HB with Met793) and the hydrophobic pocket (via hydrophobic bonds with Leu788, Ile759 and Lys745), respectively.

### 2.3. MD Simulations

**Figure 11:**
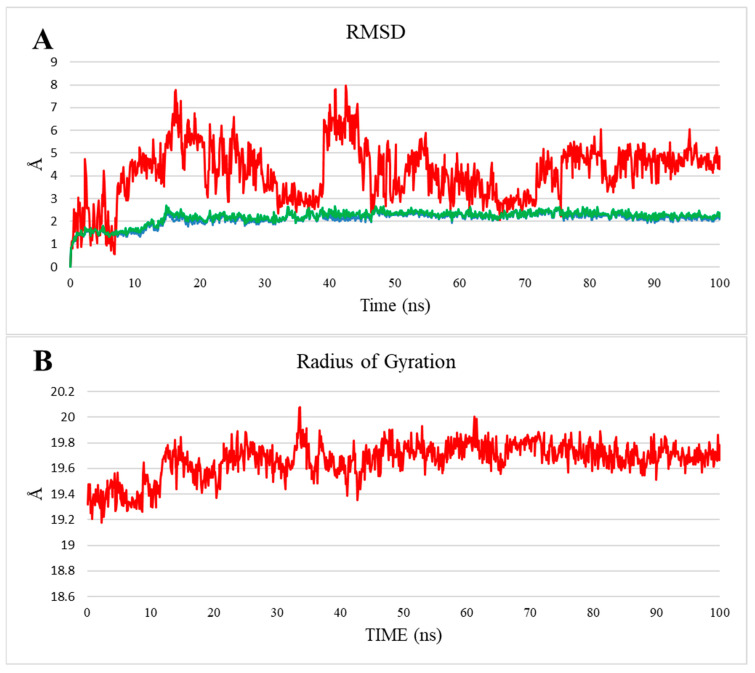

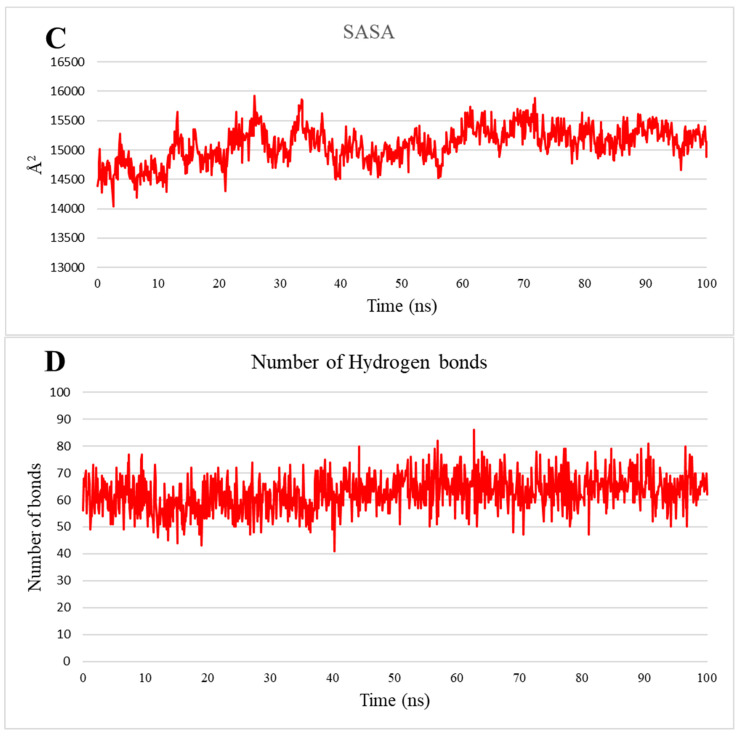

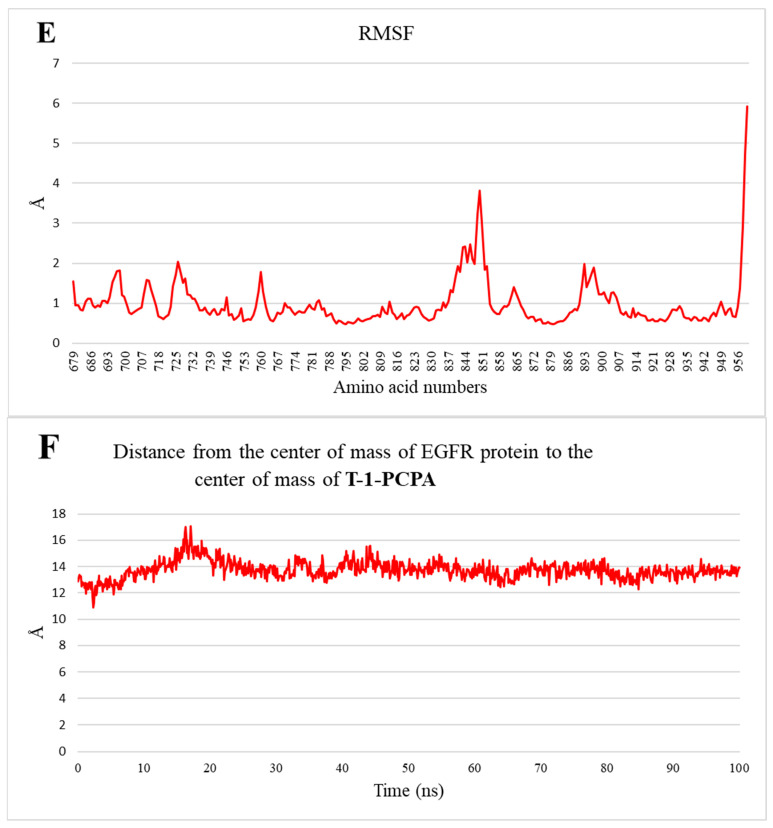
Shows the different measurements calculated from the trajectory. (**A**) RMSD: EGFR is the blue curve; **T-1-PCPA** is the red curve; and EGFR-**T-1-PCPA** complex is the green curve. (**B**) RoG, (**C**) SASA, (**D**) Change in Number of H-bonds, (**E**) RMSF and (**F**) Center of Mass distance between the **T-1-PCPA** and EGFR.

To verify the molecular docking’s results, deep MD simulations studies were performed on the EGFR-**T-1-PCPA** complex over 100 ns. Analysis of the production run indicates that the EGFR-**T-1-PCPA** system exhibits steady behavior with large **T-1-PCPA** fluctuations. The RMSD plot (Figure 11A) reveals a consistent average for the EGFR protein (blue) and the EGFR-**T-1-PCPA** complex (green) with values around 2.2 Å after roughly the first 15 ns. The RMSD of **T-1-PCPA** (red), on the other hand, exhibits significant oscillations. The values show a repeated trend for the intervals from 10 ns to 40 ns and from 40 ns to 70 ns before stabilizing in the last 30 ns around an average of 4.4 Å. Similar trends may be seen in the RoG (Figure 11B) and SASA (Figure 11C). The two measurements exhibit consistent averages of 19.7 Å and 15,157 Å^2^ after the first 15 ns, respectively (Figure 11). D of H-bonds reveals a steady variation with an average of approximately 63 bonds. The RMSF plot (Figure 11E) indicates relatively little variation (less than 2 Å) for the amino acids, except for the Y845:P853 and the C-terminal, which reach a maximum value of 3.8 Å and 6 Å, respectively. **T-1-PCPA** maintained an average distance from the EGFR center of mass of 13.7 Å throughout the simulation. This demonstrates that the significant RMSD variations are brought on by **T-1-PCPA**’s changing conformation within the binding pocket.

### 2.4. MM-GBSA

**Figure 12:**
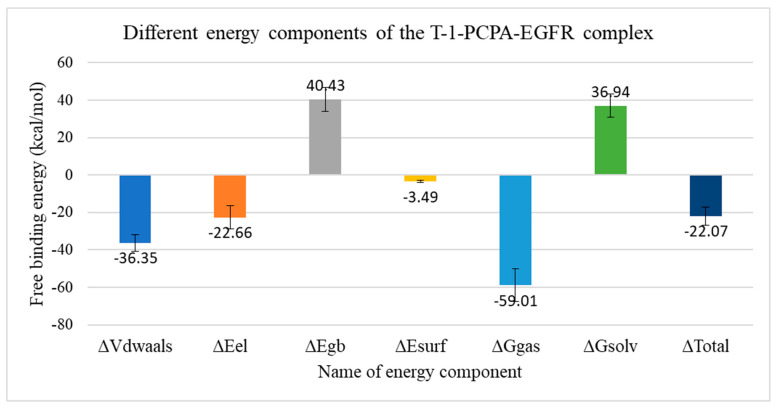
The different energetic components of MM-GBSA and their average values for the EGFR-**T-1-PCPA** complex. Bars represent the standard deviations.

**Figure 13:**
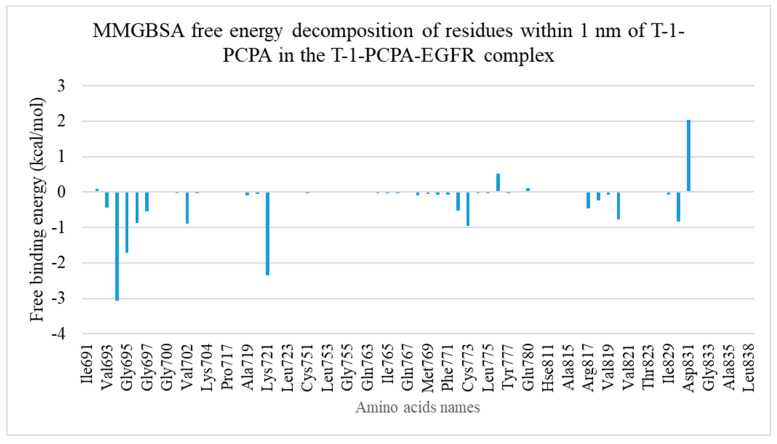
The binding free energy decomposition of the EGFR-**T-1-PCPA** complex.

Another advanced structure-based computational technique was applied to the EGFR-**T-1-PCPA** complex to confirm its stability. The different elements that contribute to the binding are displayed in the binding free energy analysis of the EGFR-**T-1-PCPA** complex using MM-GBSA (Figure 12). The average value of the overall binding for **T-1-PCPA** is −22.07 Kcal/Mol with average values for the van der Waals and electrostatic interactions around −36.35 Kcal/Mol and −22.66 Kcal/Mol, respectively. Figure 13 represents the decomposition analysis, revealing which amino acids are closest to **T-1-PCPA** and contribute most to the interaction. The amino acids that contribute with a value greater (less) than −1 Kcal/Mol are Leu694 (−3.06 Kcal/Mol), Gly695 (−1.7 Kcal/Mol) and Lys721 (−2.35 Kcal/Mol). Interestingly, there is one amino acid with a positive contribution to the binding energy with an average value of +2.03 Kcal/Mol.

### 2.5. PLIP

**Figure 14:**
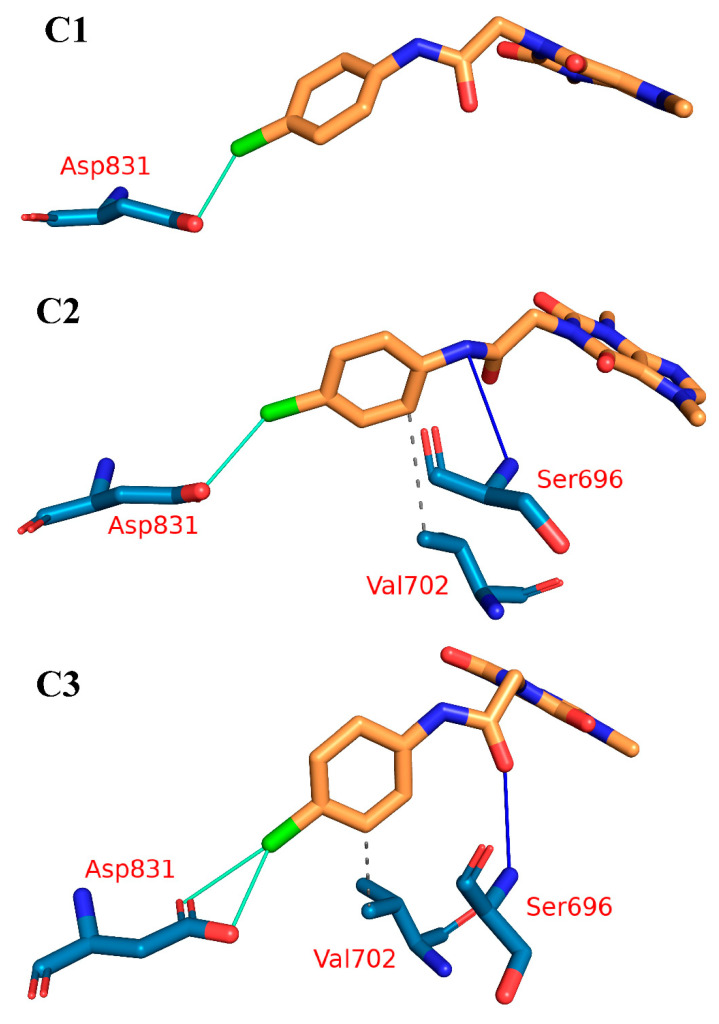
The types and number of different interactions in the EGFR-**T-1-PCPA** complex. H-bond: solid blue lines, hydrophobic interaction: dashed grey lines, halogen bonds: green solid lines, amino acids: blue sticks and **T-1-PCPA**: orange sticks.

Protein-ligand Interaction Profiler (PLIP) study was achieved to look closer at the stability as well as the changes that occur in the EGFR-**T-1-PCPA** complex through the simulation study. The obtained trajectory of the EGFR-**T-1-PCPA** complex was then clustered to provide a representative frame for each cluster. The elbow method was used to automatically choose the number of clusters, as described in the methodologies section, and this resulted in three clusters. The PLIP website was used to determine the number and types of interactions between **T-1-PCPA** and the EGFR protein for each cluster representative (Table 2). In comparison to the 4 halogen interactions between Asp831 and the chloride atom among all cluster representatives, the hydrophobic and HB interactions have two interactions in two out of the three cluster representatives. In addition to obtaining the interaction types and numbers from PLIP, it also generates a .pse file to see the 3D conformation of **T-1-PCPA** and its interaction with the protein (Figure 14).

### 2.6. ADMET Profiling Study

**Figure 15:**
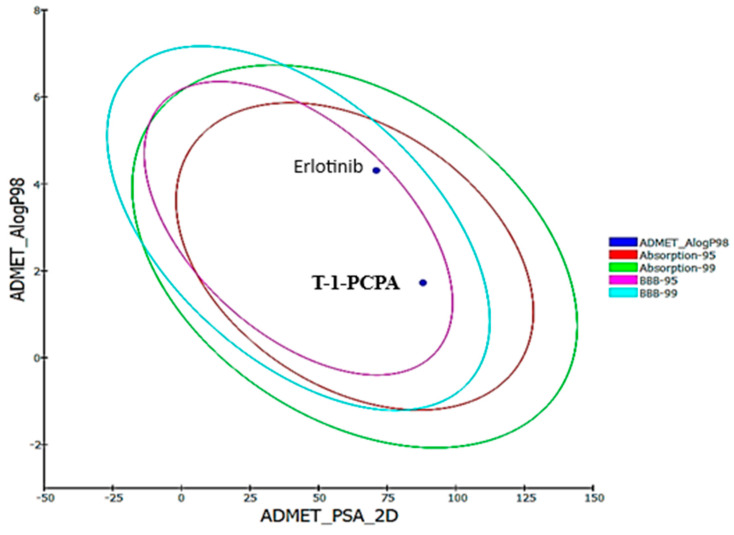
Computational calculations of the ADMET descriptors of **T-1-PCPA** and erlotinib. The two blue points represent **T-1-PCPA** and erlotinib. The ellipses represent intestinal absorption at confidence limits of 95% (red) and 99% (green); blood–brain barrier (BBB) at confidence limits of 95% (pink) and 99% (turquoise). **T-1-PCPA** lies inside the four ellipses and explains the high degrees of likeness of **T-1-PCPA**.

In order to avoid late withdrawal of a new compound from the market, it is imperative to examine the ADMET properties of the new compounds at an early stage of drug development [35]. ADMET is an acronym that stands for absorption, distribution, metabolism, excretion and toxicity, and while there are several in vitro studies that can examine the ADMET properties, computational studies are more beneficial for many reasons including time, cost, effort, as well as the strict rules that regulate in vivo experiments [36]. The ADMET outputs of **T-1-PCPA** against erlotinib (Figure 15) expressed a high degree of drug-likeness. In detail, **T-1-PCPA** showed a very low computed ability to pass the blood-brain barrier (BBB). Also, **T-1-PCPA** was predicted to be non-hepatotoxic and non-inhibitor for the cytochrome P-450 (CYP2D6). Additionally, as Table 3 shows, **T-1-PCPA** expressed good levels of aqueous solubility as well as human intestinal absorption. Interestingly, the computed ADMET descriptors of erlotinib were in agreement with the reported studies as it exhibited oral bioavailability of about 60% [37]. Also, about 93% of erlotinib is bound to the plasma proteins [38]. Additionally, several hepatorenal syndrome and hepatic failure cases (including fatalities) were reported during use of erlotinib [39]. Finally, the potentialities of erlotinib to pass the BBB and exert anticancer activities were reported [40,41,42].

### 2.7. In Silico Toxicity Studies

Secondly, in silico methods have played an essential role in predicting toxicity in drug development in terms of avoiding the strict ethical regulations, resources exhaustion and the time-delay involved in the usual in vitro and in vivo experiments [43,44]. In silico toxicity prediction relies upon the Structure-Activity Relationship (SAR)-predictive toxicity, in which the software compares the basic chemical structural descriptors of the studied molecule/s against the structures of thousands of molecules that have been reported as safe or toxic [45]. The toxicity parameters have been estimated computationally from toxicity models created in Discovery studio software, and the employed models are: FDA Rodent Carcinogenicity in Mouse-male (FDA-C-MM), Carcinogenic Potency TD_50_ in Rats (C- TD_50_-R), Ames Mutagenicity (A-M), Rat Maximum Tolerated Dose, Feed, (R-MTD-F), Rat Oral LD_50_ (R-O-LD_50_), Rat Chronic LOAEL (R-C- LOAEL), skin and ocular irritancy. As Table 4 shows, the studies indicated the general safety of **T-1-PCPA** comparing erlotinib.

### 2.8. Chemistry

**Scheme 1:**
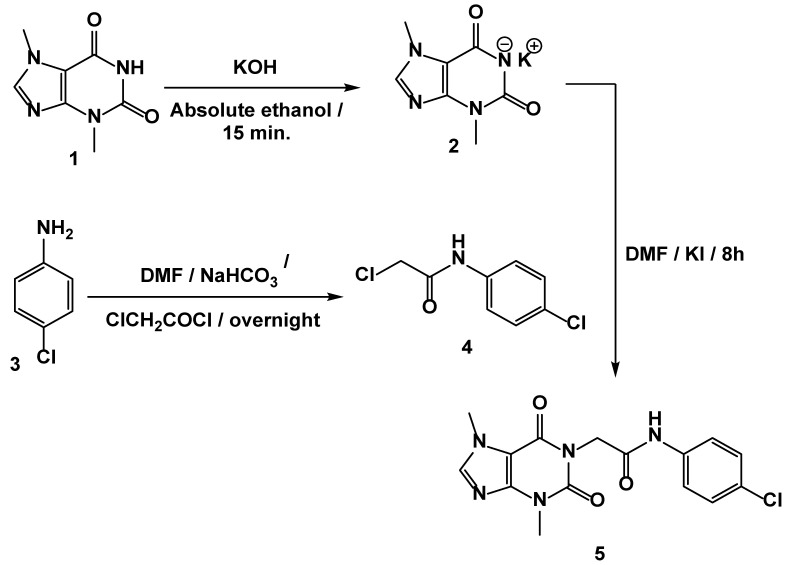
The semi-synthesis of **T-1-PCPA**.

Scheme 1 illustrates the synthetic route used in this study to produce **T-1-PCPA**. Theobromine (**1**) was first transformed into its potassium salt (**2**) through refluxing with alcoholic KOH. Then the formed potassium salt was refluxed with 2-chloro-*N*-(4-chlorophenyl)acetamide (**4**) in DMF/KI mixture to produce the final **T-1-PCPA**. The chemical structure of **T-1-PCPA** was confirmed through different spectroscopic techniques (IR, ^1^H NMR, ^13^C NMR), which were provided in detail in the Supplementary Data.

### 2.9. Biology

#### 2.9.1. In Vitro EGFR Inhibition

**Figure 16:**
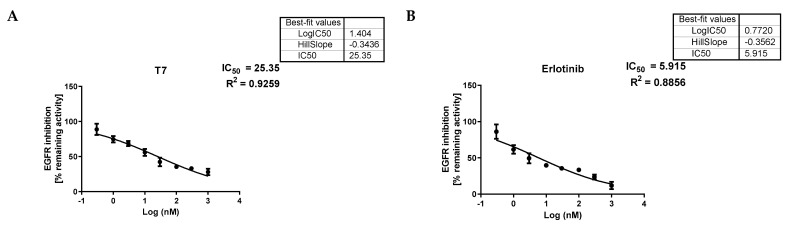
In vitro inhibition of **T-1-PCPA** (**A**) and Erlotinib (**B**) against EGFR^WT^.

To examine the performed design as well as the conducted great computational results, the potentiality of **T-1-PCPA** against EGFR^WT^ protein was investigated in vitro. Verify firmly inhibited EGFR protein with an IC_50_ value of 25.35 nM (Table 5 and Figure 16) comparing erlotinib’s value (5.90 nM).

#### 2.9.2. Cytotoxicity

**Figure 17:**
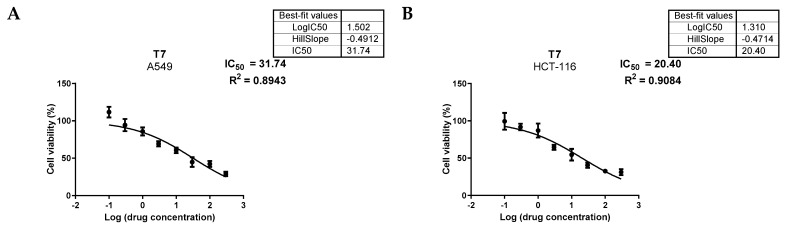

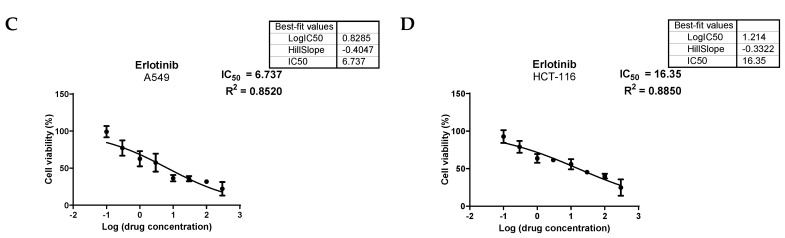
In vitro anti-proliferative effects of **T-1-PCPA** against A54 (**A**) and HCT-116 (**B**) cancer cell lines, comparing erlotinib’s activities against A54 (**C**) and HCT-116 (**D**) cancer cell lines.

The cytotoxicity of **T-1-PCPA** was investigated in vitro against A549 and HCT-116 malignant cell lines comparing erlotinib as a reference drug. The two types of cancer cells were selected because of the reported high level of EGFR expression in both of them [46,47]. Table 6 and Figure 17 show that **T-1-PCPA** expressed strong anticancer properties. In detail, **T-1-PCPA** inhibited the aforementioned cancer cells with IC_50_ values of 31.74 and 20.40 µM, respectively, comparing Erlotinib that showed IC_50_ values of 6.73 and 16.35 µM, respectively.

## 3. Conclusions

As an EGFR inhibitor, a theobromine derivative (**T-1-PCPA**) was designed to have essential structural properties that are characteristic of EGFR inhibitors. At first, the DFT calculations optimized the 3D structure and expected a high level of reactivity. In addition to molecular docking, six MD (over 100 ns), two MM-GBSA, and PLIP experiments confirmed the anti-EGFR potentiality of **T-1-PCPA**. ADMET analysis indicated safety as well. According to the in vitro results, **T-1-PCPA** displayed inhibitory effects against EGFR with an IC_50_ value of 25.35 nM. Although **T-1-PCPA** displayed an IC_50_ value of 31.74 µM against A549, which was higher than the IC_50_ value of erlotinib (6.73 µM), **T-1-PCPA**’s cytotoxic effect against HCT-116 cell lines was too close (20.40 µM) to that of erlotinib (16.35 µM). Our team proudly presents the lead compound, **T-1-PCPA**, to all scientists in the world as a key lead anticancer drug targeting the EGFR kinase for further biological evaluation and or chemical modifications.

## 4. Experimental

### 4.1. In Silico Studies

#### 4.1.1. DFT

Gaussian 09 and GaussSum3.0 programs were utilized. An extensive explanation is presented in the Supplementary Materials.

#### 4.1.2. Docking Studies

The molecular docking of **T-1-PCPA** was carried out by MOE2014 software [48]. An extensive explanation is presented in the Supplementary Materials.

#### 4.1.3. M D Simulations

CHARMM-GUI web server and GROMACS 2021 were utilized as an MD engine [49,50]. An extensive explanation is presented in the Supplementary Materials.

#### 4.1.4. MM-GBSA

The Gmx_MMPBSA package was utilized [51]. An extensive explanation is presented in the Supplementary Materials.

#### 4.1.5. ADMET Studies

ADMET profile of **T-1-PCPA** was carried out by Discovery Studio 4.0 [52]. An extensive explanation is presented in the Supplementary Materials.

#### 4.1.6. Toxicity Studies

The toxicity profile of **T-1-PCPA** was carried out by Discovery Studio 4.0. An extensive explanation is presented in the Supplementary Materials.

### 4.2. Synthesis of T-1-PCPA

An extensive explanation is presented in the Supplementary Materials.

### 4.3. Biological Studies

#### 4.3.1. In Vitro EGFR Inhibition

Human EGFR ELISA kits were utilized. Supplementary Materials show a comprehensive explanation.

#### 4.3.2. In Vitro Antiproliferative Activity

MTT procedure was performed. Supplementary Materials show a comprehensive explanation.

## Figures and Tables

**Table 1:** The calculated global reactivity indices and energetic parameters for **T-1-PCPA**.

IP	EA	μ (eV)	χ (eV)	η (eV)	σ (eV)	ω (eV)	Dm (Debye)	TE (eV)	∆N_max_	∆E (eV)
−6.206	−1.829	−4.017	4.017	2.188	0.457	17,658	8.625	−41,906.8	1.836	−17,658

**Table 2:** The number and types of interactions in the EGFR-**T-1-PCPA** complex detected from the PLIP webserver.

Cluster No.	C1	C2	C3
No. of halogen bonds	1	1	2
Amino acids in EGFR	Asp831	Asp831	Asp831(2)
No. of hydrogen bonds	0	1	1
Amino acids in EGFR	None	Ser696	Ser696
No. of hydrophobic interactions	0	1	1
Amino acids in EGFR	None	Val702	Val702

**Table 3:** Computational calculations of the ADMET descriptors of **T-1-PCPA** and erlotinib.

Comp.	BBB	Solubility	Absorption	Hepatotoxicity	CYP2D6	PBB
**T-1-PCPA**	Very low	Good	Good	Non hepatotoxic	Non inhibitor	Less than 90%
Erlotinib	High	Low		Hepatotoxic		More than 90%

**Table 4:** In silico toxicity studies of **T-1-PCPA** and erlotinib.

Comp.	FDA-C-MM	C- TD_50_-R (mg/kg/day)	A-M	R-MTD-F *	R-O-LD_50_ *	R-C- LOAEL *	Skin Irritancy	Ocular Irritancy
**T-1-PCPA**	Non-Carcinogen	12.8515	Non-Mutagen	0.0347317	1.75856	0.0108505	Non-Irritant	Mild
Erlotinib		8.05746		0.0827884	0.662169	0.0359487		

* g/kg.

**Table 5:** IC_50_ values of **T-1-PCPA** and Erlotinib against EGFR Kinase.

Comp.	EGFR IC_50_ (nM)
**T-1-PCPA**	25.35
Erlotinib	5.90

**Table 6:** In vitro anti-proliferative activities of **T-1-PCPA** against A549 and HCT-116 cell lines.

Comp.	In Vitro Cytotoxicity IC_50_ (µM) ^a^	
	A549	HCT-116
**T-1-PCPA**	31.74 ± 1.55	20.40 ± 1.22
Erlotinib	6.73 ± 0.65	16.35 ± 1.01

**^a^** Data are presented as the mean of the IC_50_ values from three different experiments.

## Data Availability

Data are available with corresponding authors upon request.

## Supplementary Materials

The details of experimental part as well as the spectral data (^1^H NMR, ^13^C NMR, Ms, IR, and elemental analysis of **T-1-PCPA** can be downloaded at: https://www.mdpi.com/article/10.3390/life13010191/s1).
