# Supplementary material for: A New Anticancer Semisynthetic Theobromine Derivative Targeting EGFR Protein: CADDD Study
Source: Life (Basel). 2023 Jan 9;13(1):191. doi: 10.3390/life13010191 (PMC9867533; doi:10.3390/life13010191)
Supplement: Supplementary file 1 [file life-13-00191-s001.zip › life-2049262-SI.pdf]

Supplementary file

A New Anticancer Semisynthetic Theobromine Derivative Targeting EGFR Protein: CADDD Study

Ibrahim H. Eissa [1,*], Reda G. Yousef [1], Hazem Elkady [1], Aisha A. Alsfouk [2], Bshra A. Alsfouk [2], Dalal Z. Husein [3], Ibrahim M. Ibrahim [4], Eslam B. Elkaeed [5] and Ahmed M. Metwaly [6,7,*]

[1] Pharmaceutical Medicinal Chemistry & Drug Design Department, Faculty of Pharmacy (Boys), Al-Azhar University, Cairo 11884, Egypt
[2] Department of Pharmaceutical Sciences, College of Pharmacy, Princess Nourah bint Abdulrahman University, Riyadh 11671, Saudi Arabia
[3] Chemistry Department, Faculty of Science, New Valley University, El-Kharja 72511, Egypt
[4] Biophysics Department, Faculty of Science, Cairo University, Cairo 12613, Egypt
[5] Department of Pharmaceutical Sciences, College of Pharmacy, AlMaarefa University, Riyadh 13713, Saudi Arabia
[6] Pharmacognosy and Medicinal Plants Department, Faculty of Pharmacy (Boys), Al-Azhar University, Cairo 11884, Egypt
[7] Biopharmaceutical Products Research Department, Genetic Engineering and Biotechnology Research Institute, City of Scientific Research and Technological Applications (SRTA-City), Alexandria 21934, Egypt
* Correspondence: authors: ibrahimeissa@azhar.edu.eg (I.H.E.); ametwaly@azhar.edu.eg (A.M.M.)

Content

**Method**	**DFT**
	**Molecular Docking**
	**MD Simulations**
	**MM-GBSA**
	**ADMET studies**
	**Toxicity studies**
	**Synthesis**
	***In vitro* assays**
**Spectral Data**	**IR of T-1-PCPA**
	**^{1}H and ^{13}C NMR of T-1-PCPA**
	**Mass and Elemental analysis of T-1-PCPA**

Method

- **Density Function Theory (DFT) calculations**

The Gaussian 09 program was used to perform the quantum chemistry calculations using the DFT method. GaussianView5 was used to display all of the data files. The density function theory (DFT) at 6-311G++(d,p) basis set/B3LYP approach was utilized to optimize organic chemical structure of the compound under investigation and Chem3D 15.0 software was used to create the original chemical structures. Both the Total Electron Density (TED) and the Electrostatic Surface (ESP) maps were examined at the same theoretical level. GaussSum3.0 software was used to compute and evaluate the totaldensity of state (TDOS) for the optimized log file.

Equations of Koopmans' theory: The chemical potential (μ), maximal charge acceptance (ΔN_{max}), global hardness (η), energy change (ΔE), electronegativity (χ), the global softness (σ), electrophilicity index (ω), ionization potential (IP) and electron affinity (EA)

$IP = -E_{\text{HOMO}}$

$EA = -E_{\text{LUMO}}$

$\mu=$

$(IP+EA)/2$

$\eta= (IP-EA)$

$\chi= - \eta$

$\omega= \mu^2/(2$

$\eta)$ $\sigma= 1/$

η

$\Delta N= -(\mu/\eta)$

$\Delta E = -\omega$

$E_{gap}= E_{\text{LUMO}}-E_{\text{HOMO}}$

- **Molecular Docking studies**

Crystal structure of wild ($EGFR^{WT}$; PDB: 4HJO) and mutant ($EGFR^{T790M}$; PDB: 3W2O) types of EGFR-TK were obtained from Protein Data Bank. The docking investigation was accomplished using MOE2014 software. At first, the crystal structure of EGFR was prepared by removing water molecules. Only one chain was retained besides the co-crystallized ligand (erlotinib). Then, the selected chain was protonated and subjected to the minimization of the energy process. Next, the active site of the target protein was defined.

Structures of the synthesized compound and erlotinib were drawn using ChemBioDraw Ultra 14.0 and saved as MDL-SD format. Such a file was opened using MOE to display the 3D structures which were protonated and subjected to energy minimization. Formerly, validation of the docking process was performed by docking the co-crystallized ligand against the isolated pocket of the active site. The produced RMSD value indicated the validity of the process.Finally, docking of the tested compoundswas donethrough thedock option inserted in compute window. For each docked molecule, 30 docked poses were produced using ASE for scoring function and force field for refinement. The results of the docking process were then visualized using Discovery Studio 4.0 software.

- **Molecular Dynamic Simulation**

Molecular Dynamic Simulation:

In this study, we performed a classical molecular dynamic (MD) simulation to study the stability and the binding affinity of the protein-T-1-PCPA complex. To prepare the system, the solution builder module in the CHARMM-GUI web server was utilized to generate the necessary files. First, the complex was uploaded as a PDB file, solvated using the transferable intermolecular potential 3P (TIP3P) water model in a cubic box with a padding of 1 nm, and neutralized with Na^+ and Cl^- ions to the physiological concentration of 0.154 M of salt concentration. The CHARMM36m force field was used to parameterize the amino acids of the protein, neutralizing ions, and water molecules while the CHARMM general force field (CGenFF) tool implemented in CHARMM-GUI wasutilized to parameterize the compound T-1-PCPA. GROMACS 2021. was

utilized as an MD engine to perform the simulation with periodic boundary conditions (PBC) applied to the system. Before the production run, the system must be minimized and equilibrated therefore, a minimization step using the steepest descent algorithm with a max force set to 100 $KJ.mol^{-1}.nm^{-1}$ as a convergence criterion was initiated followed by two equilibration steps. The first equilibration step was in a constant number of atoms, constant volume, and constant temperature (NVT) ensemble while the second step was in a constant number of atoms, constant pressure, and constant temperature (NPT) ensemble. The temperature was set to 310 K and maintained using the V-rescale algorithm during the equilibration. On the other hand, the pressure was set to 1 atmospheric pressure and maintained using the Berendsen barostat. Finally, the production run was in an NVT ensemble for 100 ns and the temperature was maintained using the Nose-Hoover thermostat. In each step, the bond lengths of hydrogen-bonded atoms were constrained using LINear Constraint Solver (LINCS) algorithm. The calculation of the electrostatics was performed using Particle Mesh Ewald (PME) (9) algorithm with a cutoff of 1.2 nm. To integrate the Newtonian equations of motion, the leap-frog algorithm was used with a time step of 1 femtosecond for the equilibration steps and 2 femtoseconds for the production step. The production run was saved for every 100 picoseconds with a total of 1000 frames. Before analyzing the trajectory, the PBC was removed using the trjconv tool in GROMACS. The analysis of the production trajectory was performed using VMD TK scripts. Root mean square deviation (RMSD) for the protein alone, the compound T-1-PCPA, and the complex were measured. In addition, the root mean square fluctuation (RMSF), the solvent accessible surface area (SASA), the radius of gyration (RoG), the number of hydrogen bonds, and the distance between the center of mass of the compound T-1-PCPA and the center of mass of the protein were measured. Afterward, the trajectory was clustered using TTClust. to get a representative frame for each cluster. First,

backbone alignment was performed before determining the optimum number of clusters using the elbow method. For each representative frame, protein Ligand interaction profiler (PLIP) was used to detect the number and types of interactions.

Binding free energy calculation using MM-GBSA:

To find the binding affinity, the gmx_MMPBSA package was used with Molecular Mechanics Generalized Born Surface Area (MM-GBSA) algorithm. In addition, decomposition analysis was calculated to get the binding energies of amino acidswithin 1 nm around thecompound T-1-PCPA. The salt concentration and the method of solvation (igb) were set to 0.154 M and 5, respectively. The internal and external dielectric constants were set to 1.0 and 80.0, respectively, and other options were set as default. MM-GBSA approach is depicted in Equation 1.

$$\Delta G = < G_{complex} - G_{receptor} - G_{compound} > \quad \text{Equation 1}$$

Where < > represents the average of the enclosed free energies of the complex, he receptor, and compound over the frames used in calculation. In our approach, we used the whole trajectory (a total of 1000 frames). Different energy terms can be calculated according to Equations 2 to 6 as follows:

$$\Delta G_{binding} = \Delta H - T\Delta S \quad \text{Equation 2}$$

$$\Delta H = \Delta E_{gas} + \Delta E_{sol} \quad \text{Equation 3}$$

$$\Delta E_{gas} = \Delta E_{ele} + \Delta E_{vdW} \quad \text{Equation 4}$$

$$\Delta E_{solv} = E_{GB} + E_{SA} \quad \text{Equation 5}$$

$$E_{SA} = \gamma.SASA \quad \text{Equation 6}$$

Where:

ΔH is the enthalpy which can be calculated from gas-phase energy (E_{gas}) and solvation-free energy (E_{sol}). TΔS is the entropy contribution to the free binding energy. E_{gas} is composed of electrostatic

and van der Waals terms; E_{ele}, E_{vdW}, respectively. E_{sol} can be calculated from the polar solvation energy (E_{GB}) and nonpolar solvation energy (E_{SA}) which is estimated from the solvent-accessible surface area .

- **Preparation of the tested compounds for ADMET, and toxicity studies**

In this protocol, the general-purpose panel was utilized with the activation of the Prepare ligand option. The change ionization was switched on the true option using the Rule based as an ionization method. In Rule based task, we used the carboxylate as an acid ionization. Additionally, the primary, secondary, and tertiary amines were selected as Base ionization. The ionization enumeration option was switched on the one protomer. Under the filter smart option, we selected all options. The false option was selected for tasks Generate tautomers, generate isomers, Fix bad valencies, and parallel processing. The generate coordinates task was switched on the 3D option. Finally, the duplicate structure task was activated on the remove option.

- **ADMET studies**

ADMET descriptors (absorption, distribution, metabolism, excretion and toxicity) of the compounds were determined using Discovery studio 4.0. At first, the CHARMM force field was applied then the tested compounds were prepared and minimized according to the preparation of small molecule protocol. The ADMET descriptors that applied including models for

1. Human intestinal absorption,
2. Aqueous solubility,
3. Blood brain barrier penetration,
4. Plasma protein binding,
5. Cytochrome P450 2D6 inhibition, and
6. Hepatotoxicity.

The examined molecules filtered to select those molecules that meet the rules specified by the set of selected SMARTS® rules.

.

Running of ADMET protocol

In this protocol, after compounds preparation, the small molecules panel was utilized with the activation of the ADMET descriptors option. Then, we selected the prepared compounds as the

input ligands. Further, all the ADMET parameters (aqueous solubility, Blood brain barrier, intestinal absorption, CYP2D6, and plasma protein binding) were selected. Then, the output of the running protocol was visualized to give the ADMET chart.

- **Toxicity studies**

The toxicity parameters of the synthesized compounds were calculated using Discovery studio 4.0. Then different parameters were calculated from the toxicity prediction (extensible) protocol (TOPKAT) that evaluated the examined compounds' performance in experimental assays and animal models. TOPKAT computed and validated assessments of the toxic and environmental effects of the examined chemicals solely from their molecular structure. TOPKAT employs robust and cross-validated Quantitative Structure Toxicity Relationship (QSTR) models for assessing various measures of toxicity and utilizing the patented Optimal Predictive Space validation method to assist in interpreting the results.

The predicted models are

1. FDA rat carcinogenicity test,
2. AMES Carcinogenicity
3. Carcinogenic potentiality TD_{50} (the median toxic dose of a substance in which toxicity occurs in 50% of a species),
4. Maximum tolerated dose (MTD) in rats,
5. Oral LD_{50} in rats (the amount that kills 50% of testanimals),
6. Chronic LOAEL (Lowest-observed-adverse-effect level) in rats,
7. Ocular irritancy and
8. Skin irritancy

- **Chemistry**

General

The melting point were carried out by open capillary method on a Gallen kamp Melting point apparatus. The infrared spectra were recorded on pye Unicam SP 1000 IR spectrophotometerusing potassium bromide disc technique. Proton magnetic resonance ^{1}HNMR spectra were recorded on a Bruker 400 Megahertz-nuclear magnetic resonance (400 MHZ-

NMR) spectrophotometer. Carbon-13 (C13) nuclear magnetic resonance (^{13}CNMR) spectra were recorded on a Bruker 100 Megahertz-nuclear magnetic resonance (100 MHZ-NMR) spectrophotometer. Tetramethylsilane (TMS) was used as internal standard and chemical shifts were measured in δ scale one part per million (ppm). The reactions were monitored by thin-layer chromatography (TLC) using TLC sheets precoated with UV fluorescent silica gel Merck 60 F254 plates and were visualized using ultraviolet (UV) lamp and different solvents as mobile phases.

General procedure for the synthesis ofT-1-PCPA

The potassium 3,7-dimethyl-3,7-dihydro-1*H*-purine-2,6-dione **2** (0.001 mol) in dry DMF (10 mL) solution was mixed with 2-chloro-*N*-(4-chlorophenyl)acetamide **4** (0.001 mol), and the mixture was heated in a water bath for 5 h. Workup of the reaction involved addition of ice water while stirring. The formed powder was filtered and crystallized from methanol to produce the final **T-1-PCPA**.

***N*-(4-Chlorophenyl)-2-(3,7-dimethyl-2,6-dioxo-2,3,6,7-tetrahydro-1*H*-purin-1-yl)acetamide**

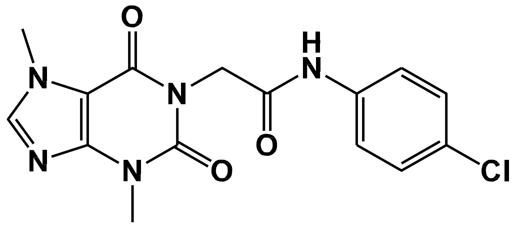

Off-white crystal (yield, 84 %); m. p. = 257-259 °C; IR (KBr) *ν* cm^{-1}: 3247 (NH), 3039 (CH aromatic), 2969, 2924 (CH aliphatic), 1714, 1661 (C=O); ^{1}H NMR (400 MHz, DMSO-d_6) δ 10.44 (s, 1H), 8.09 (s, 1H), 7.60 (d, J = 8.7 Hz, 2H), 7.38 (d, J = 8.8 Hz, 2H), 4.68 (s, 2H), 3.90 (s, 3H), 3.44 (s, 3H); ^{13}C NMR (101 MHz, DMSO-d_6) δ 166.39, 154.62,151.34, 148.98, 143.75, 138.19, 129.19, 127.37, 121.05, 107.04, 43.87, 33.69, 29.92. For $C_{15}H_{14}ClN_5O_3$ (347.76).

- **Biological evaluations**

***1-In vitro* EGFR kinaseassay**

The synthesized compound was estimated for their *in vitro* inhibition on human EGFR in MCF- 7 cell line; using ELISA kit. Firstly, a plate was used for the assay had been coated by an antibody specific for human EGFR enzyme, Erlotinib was nominated as a standard EGFR inhibitor. Both standard and sample were added to the wells and incubated overnight at 4 °C, then washed. The

biotinylated antibody was supplemented and further incubated for 1 h at room temperature. The unreacted, liberated antibody was then washed; followed by addition of HRP-conjugated streptavidin and incubated for 45 min at room temperature. Wells were washed and a TMB substrate solution was added and kept at room temperature for 30 min. Finally, the stop solution was added, and the intensity of the color produced was measured at 450 nm. Concentration-inhibition response curve was established by GraphPad Prism 5.0. The IC_{50} value was calculated as the concentration at which 50% of the cells could survive in comparison to erlotinib

2- Mammalian cell lines culture

A549 and HCT 116 cell lines were cultured on DMEM media. The cultured media were supplemented with 200 mM L-glutamine, 10.0% fetal bovine serum (Lonza), and 1.0% penicillin/streptomycin. Cells were seeded into 25.0 cm tissue culture flasks and incubated at 37°C in a 5.0% CO_2 incubator for 24 h or till confluency.

3- ***In-vitro* anticancer activity**

Anticancer activities of the tested compounds against A549 and HCT 116 cell lines were quantified using MTS assay kit (Promega) as described by the Manufacturer.

Spectral Data

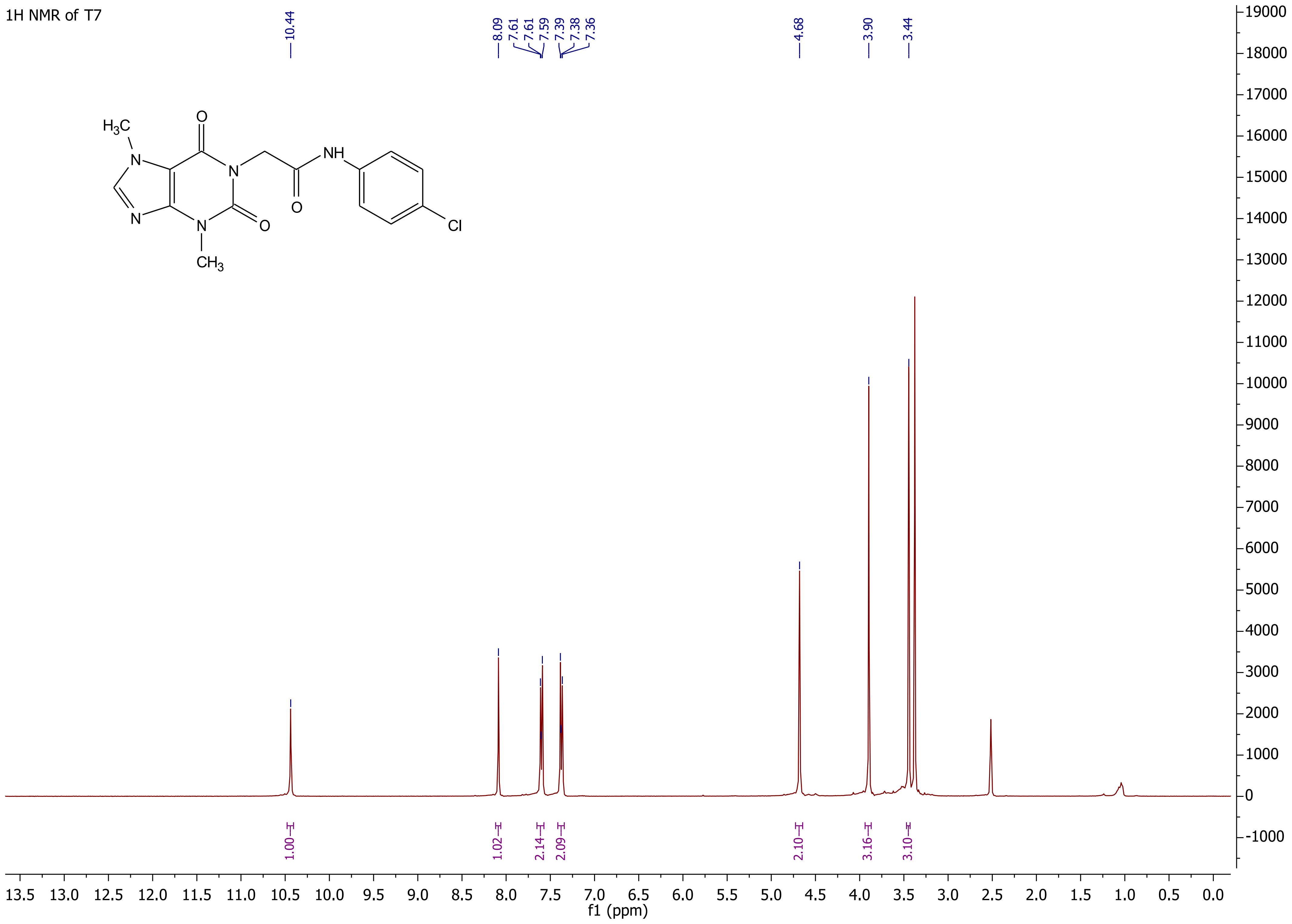

1H NMR of T7
10.44
8.09
7.61
7.61
7.59
7.39
7.38
7.36
4.68
3.90
3.44
H3C
O
N
N
NH
N
N
O
O
CH3
Cl
1.00
1.02
2.14
2.09
2.10
3.16
3.10
f1 (ppm)

1H NMR of T7

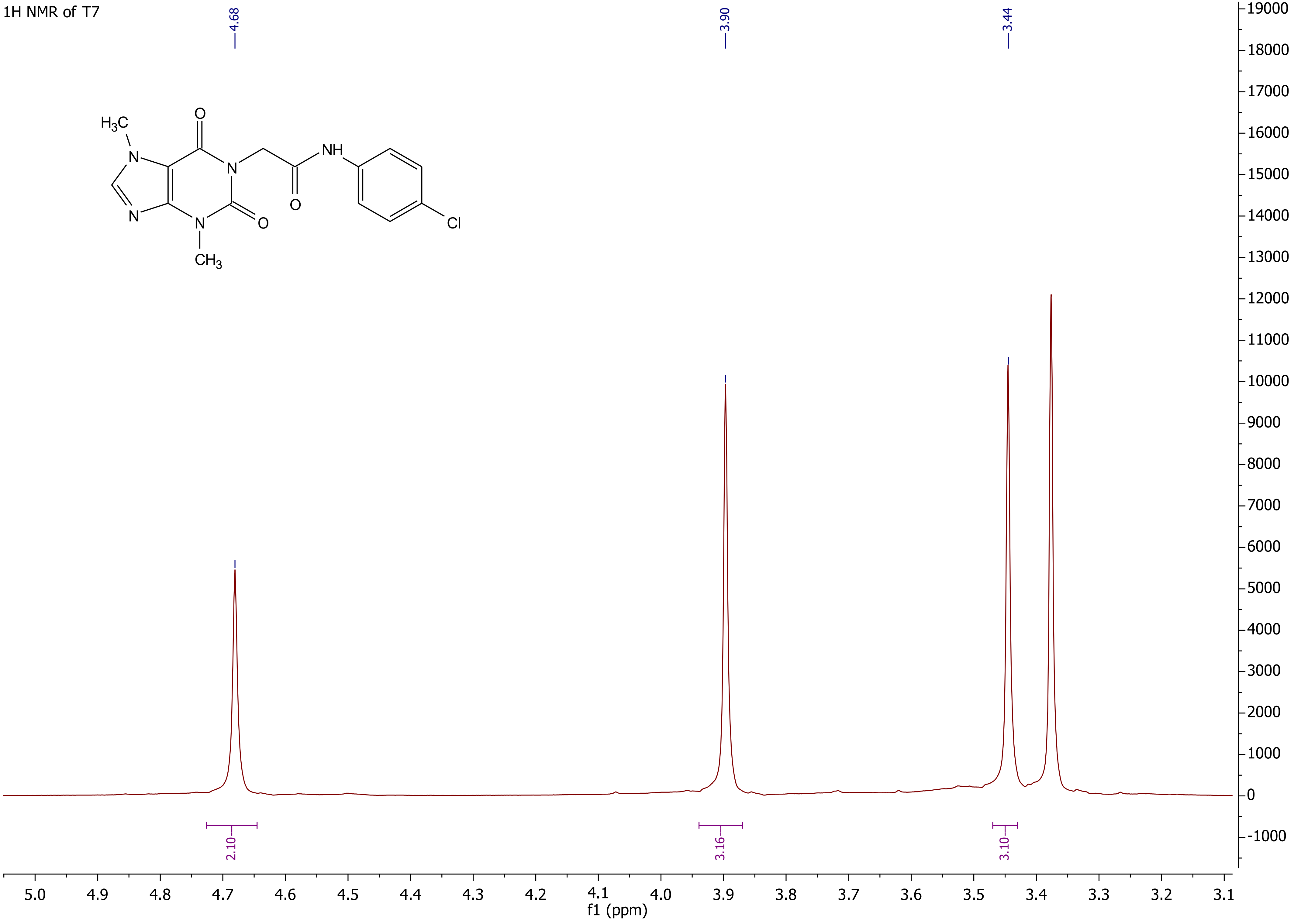

4.68
3.90
3.44
H3C
O
N
N
NH
N
O
O
N
Cl
CH3
2.10
3.16
3.10
5.0
4.9
4.8
4.7
4.6
4.5
4.4
4.3
4.2
4.1
4.0
3.9
3.8
3.7
3.6
3.5
3.4
3.3
3.2
3.1
f1 (ppm)
19000
18000
17000
16000
15000
14000
13000
12000
11000
10000
9000
8000
7000
6000
5000
4000
3000
2000
1000
0
-1000

1H NMR of T7

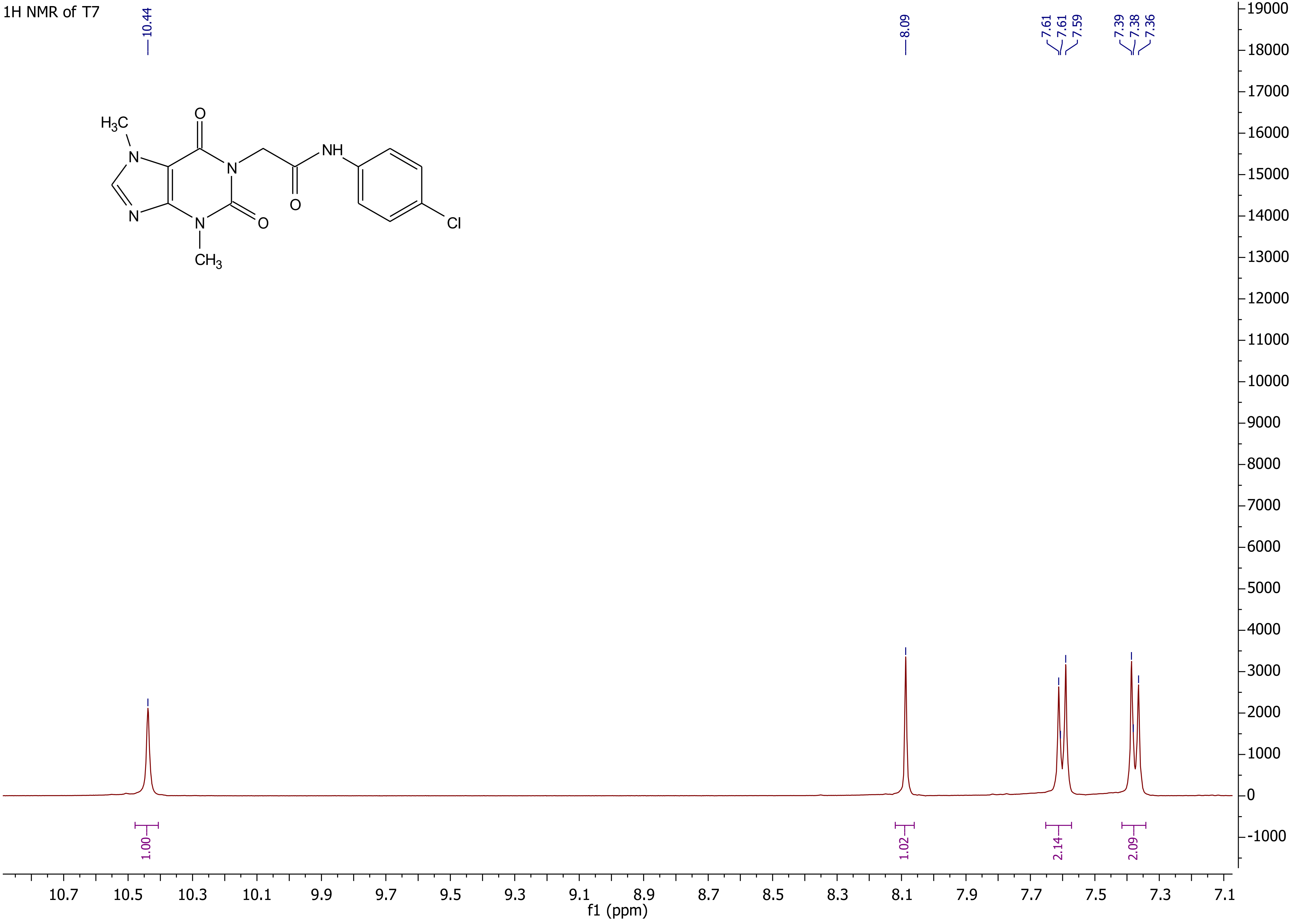

10.44
8.09
7.61
7.61
7.59
7.39
7.38
7.36
H3C
O
N
N
N
N
O
O
NH
Cl
CH3
1.00
1.02
2.14
2.09
f1 (ppm)
10.7
10.5
10.3
10.1
9.9
9.7
9.5
9.3
9.1
8.9
8.7
8.5
8.3
8.1
7.9
7.7
7.5
7.3
7.1
19000
18000
17000
16000
15000
14000
13000
12000
11000
10000
9000
8000
7000
6000
5000
4000
3000
2000
1000
0
-1000

13C NMR of T7

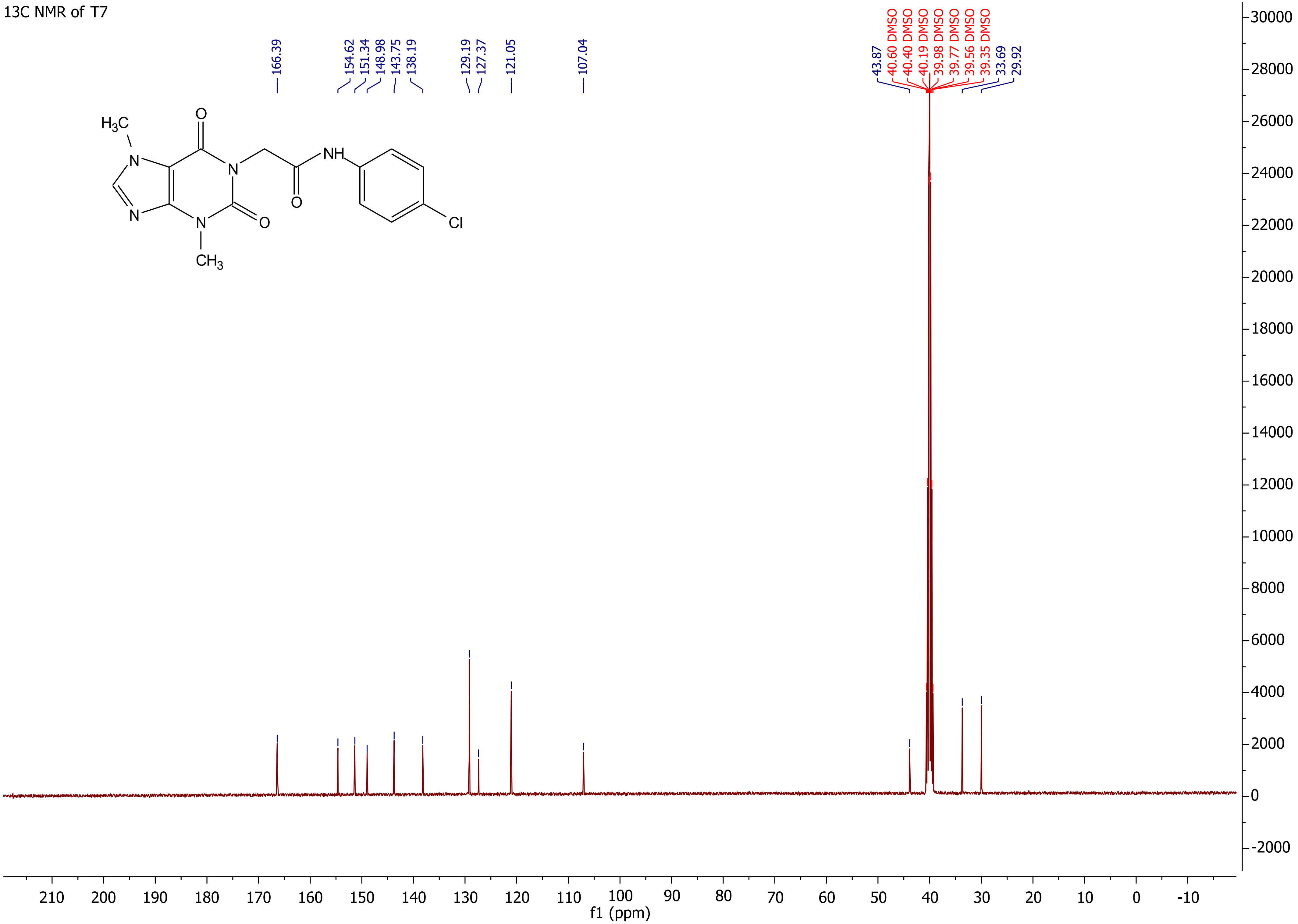

166.39
154.62
151.34
148.98
143.75
138.19
129.19
127.37
121.05
107.04
43.87
40.60 DMSO
40.40 DMSO
40.19 DMSO
39.98 DMSO
39.77 DMSO
39.56 DMSO
39.35 DMSO
33.69
29.92
H3C
N
N
N
N
O
O
O
NH
Cl
CH3
f1 (ppm)

13C NMR of T7

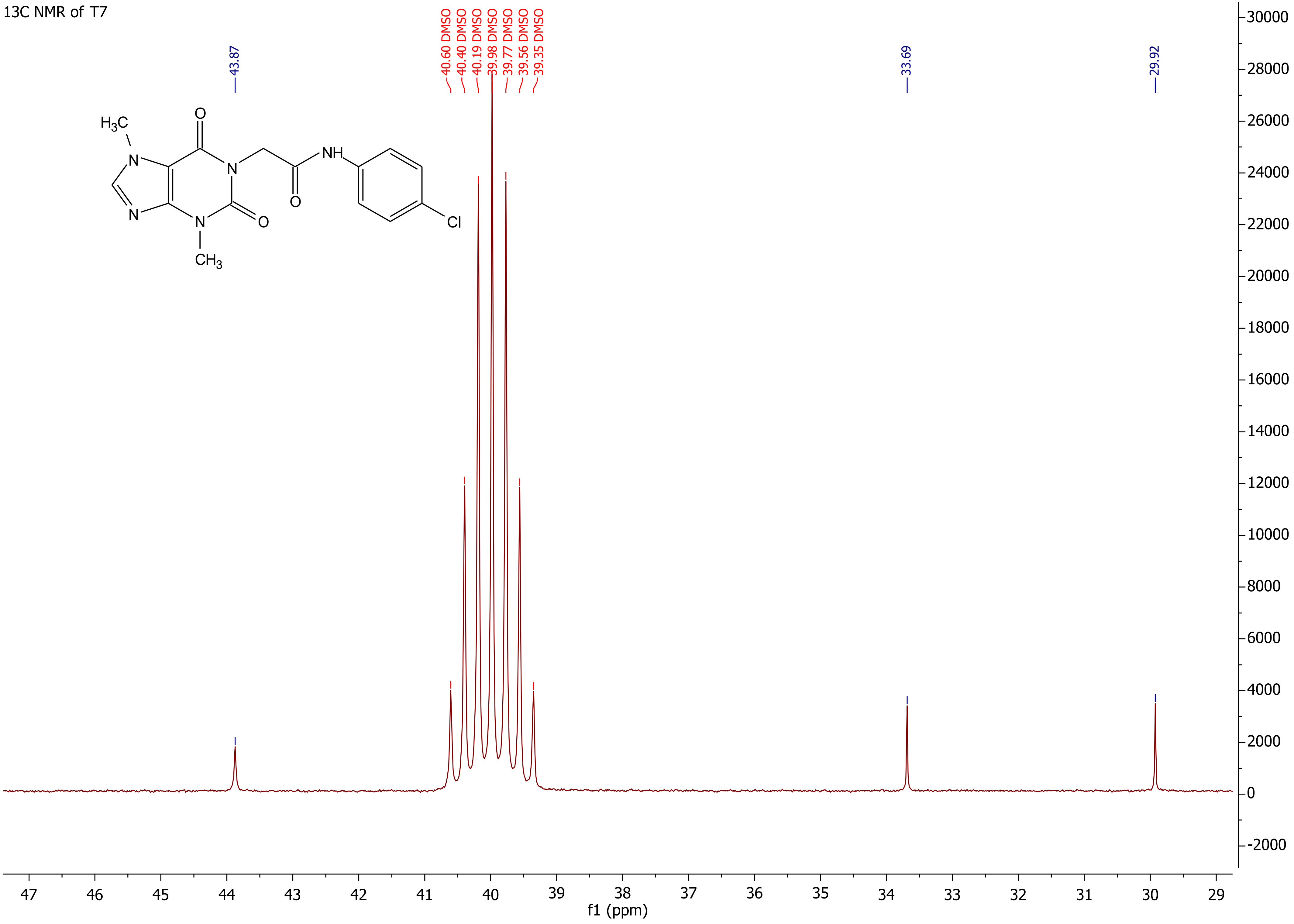

43.87
40.60 DMSO
40.40 DMSO
40.19 DMSO
39.98 DMSO
39.77 DMSO
39.56 DMSO
39.35 DMSO
33.69
29.92
H3C
O
N
N
NH
O
Cl
N
N
O
CH3
f1 (ppm)

13C NMR of T7

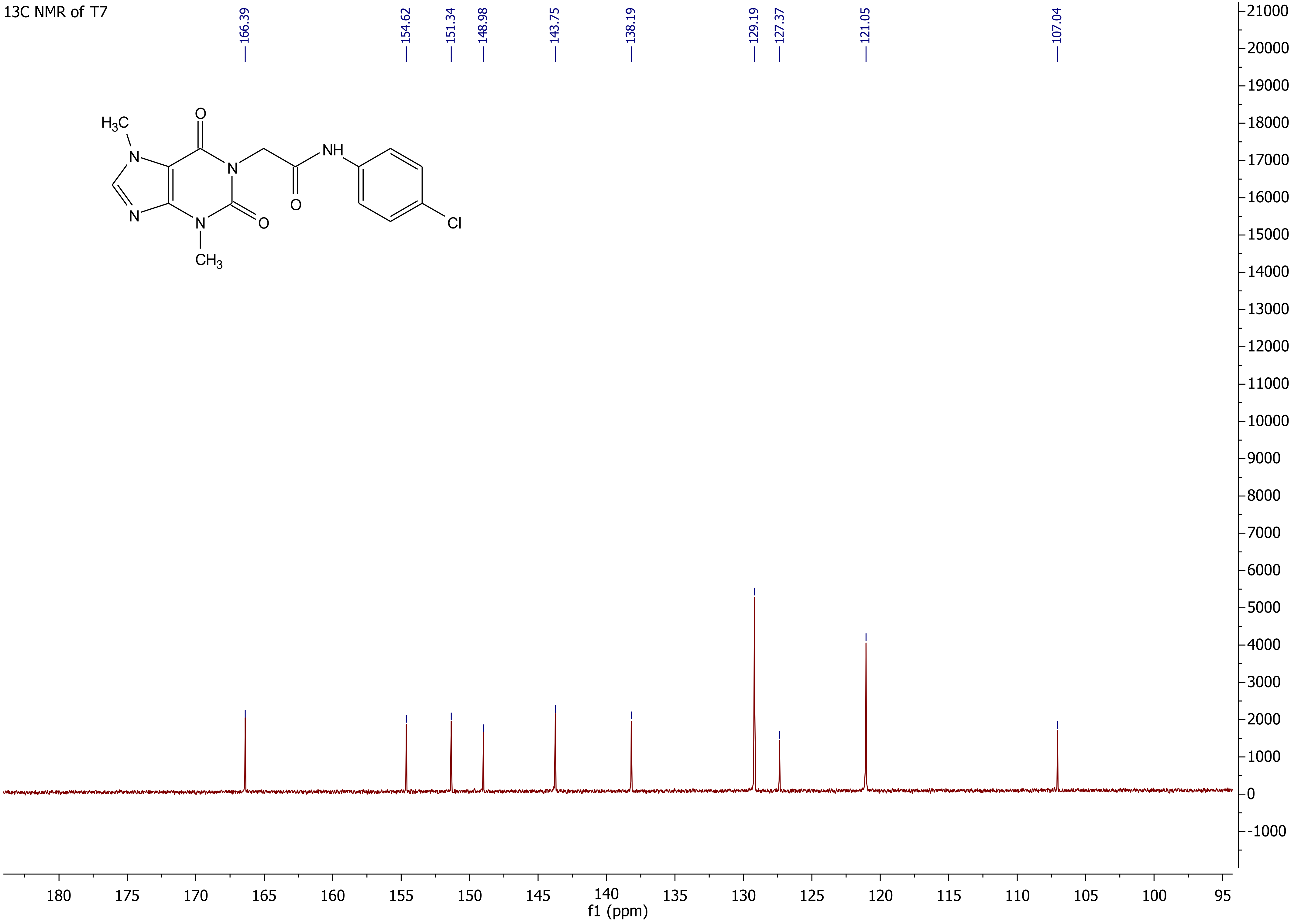

166.39
154.62
151.34
148.98
143.75
138.19
129.19
127.37
121.05
107.04
f1 (ppm)

neRe .4/zhar Universitj nolo

gional Center for Mycolo and Blotecb

Req u ester Data:

Name: Dr. Ibrahim Hasan Eissa

Authority: Faculty of Pharmacy, Al-Azhar University

Sample Data:

One sample had been submitted for elemental analysis.

Analysis Report:

Sam ple Code	C%	fl%	N%
T-7	51.74	4.72	20.28

INVESTIGATOR

r ,7/M

DIRECTOR

1 S(k.

جامعة الأزهر

المركز الإقليمي للفطريات وتطبيقاتها

AI-Azhar University Campus - Nasr City, Cairo, Egypt.
Tel: 0202 22620373 Fax : 0202 22620373
E.mail:rcmb@azhar.edu.eg
Website: http://www.azhar.edu.eg.htm * http://www.azhar.edu.eg/pages/fungi_center.htm
Facebook : RCMB AZHAR P.O. box mail : 11751 Nasr City Cairo, Egypt.

The Regional Center For mycology And Biotechnology
RCMB

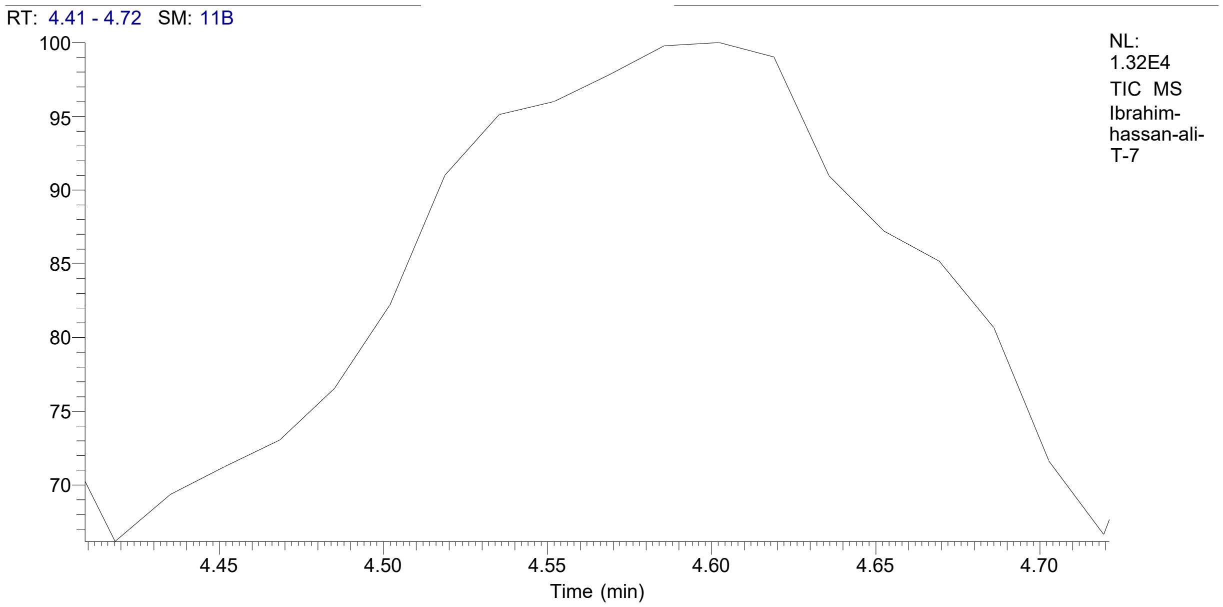

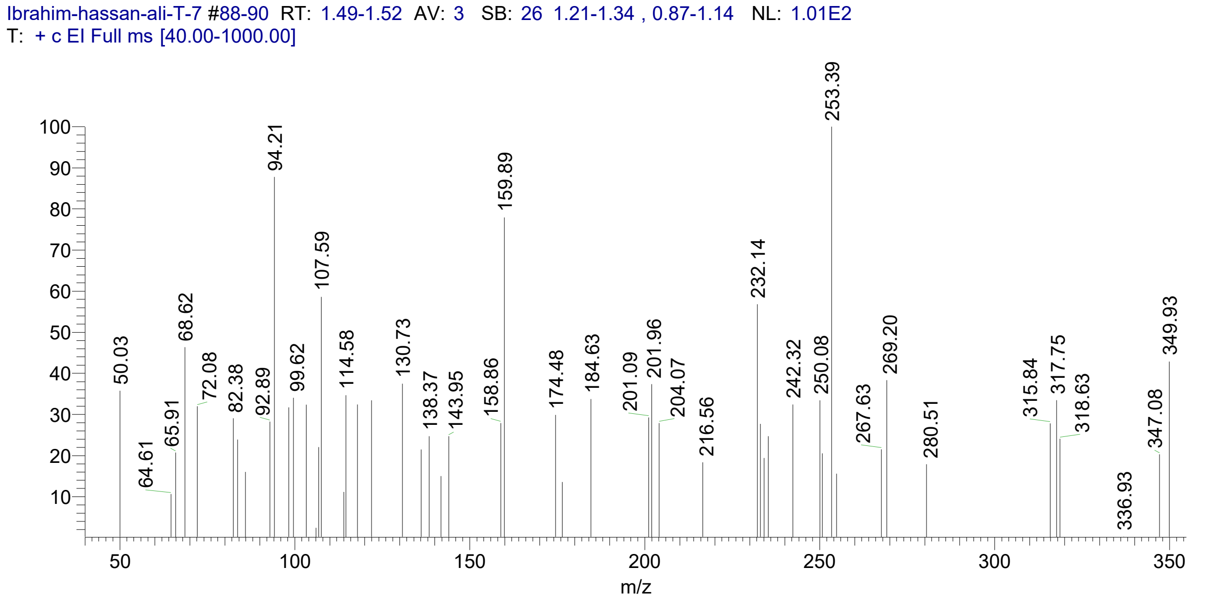

m/z	Intensity	Relative
50.03	36.0	35.77
64.61	10.7	10.66
65.91	20.9	20.71
68.62	46.7	46.38

72.08	32.3	32.02
82.38	29.3	29.06
83.60	24.1	23.91
85.90	16.2	16.03
92.89	28.5	28.26
94.21	88.4	87.77
98.27	32.0	31.74
99.62	34.4	34.09
103.20	32.6	32.37
106.04	2.5	2.45
106.82	22.2	22.01
107.59	59.1	58.66
113.99	11.3	11.17
114.58	35.0	34.71
117.85	32.7	32.46
121.92	33.7	33.46
130.73	37.8	37.50
136.09	21.7	21.51
138.37	24.9	24.69
141.75	15.1	14.98
143.95	24.9	24.68
158.86	28.1	27.90
159.89	78.5	77.93
174.48	30.1	29.89
176.40	13.7	13.59

184.63	34.0	33.74
201.09	29.5	29.31
201.96	37.7	37.37
204.07	28.1	27.92
216.56	18.5	18.35
232.14	57.3	56.87
233.00	28.0	27.74
234.07	19.5	19.40
235.25	24.9	24.75
242.32	32.7	32.46
250.08	33.7	33.48
250.67	20.7	20.54
253.39	100.8	100.00
254.80	15.7	15.60
267.63	21.7	21.53
269.20	38.7	38.37
280.51	18.0	17.86
315.84	28.1	27.88
317.75	33.7	33.48
318.63	24.3	24.11
336.93	0.3	0.33
347.08	20.5	20.30
349.93	43.2	42.90
